# Applications of MXene and its modified materials in skin wound repair

**DOI:** 10.3389/fbioe.2023.1154301

**Published:** 2023-03-13

**Authors:** Ziyan Zhang, Zhiping Qi, Weijian Kong, Renfeng Zhang, Chunli Yao

**Affiliations:** ^1^ Department of Orthopedic Surgery, The Second Hospital of Jilin University, Changchun, China; ^2^ The Second Hospital of Jilin University, Changchun, China; ^3^ Department of Dermatology, The Second Hospital of Jilin University, Changchun, China

**Keywords:** skin wound repair, MXene, biocompatibility, conductivity, antibacterial

## Abstract

The rapid healing and repair of skin wounds has been receiving much clinical attention. Covering the wound with wound dressing to promote wound healing is currently the main treatment for skin wound repair. However, the performance of wound dressing prepared by a single material is limited and cannot meet the requirements of complex conditions for wound healing. MXene is a new two-dimensional material with electrical conductivity, antibacterial and photothermal properties and other physical and biological properties, which has a wide range of applications in the field of biomedicine. Based on the pathophysiological process of wound healing and the properties of ideal wound dressing, this review will introduce the preparation and modification methods of MXene, systematically summarize and review the application status and mechanism of MXene in skin wound healing, and provide guidance for subsequent researchers to further apply MXene in the design of skin wound dressing.

## 1 Introduction

With the rapid development of society, the incidence of skin injury caused by trauma, disease and other factors in life is increasing (LeBlanc et al., 2019). Skin is an important protective organ of the human body. Maintaining its integrity can provide a physical barrier for the body to prevent the invasion of foreign harmful substances, reduce the loss of water and electrolyte, and maintain the stability of the internal environment (Dąbrowska et al., 2018; Swaney and Kalan, 2021). Therefore, it is very important to promote rapid healing of skin wound. Wound dressing can cover the surface of the wound to protect the wound, reduce the impact of external factors and stimulation on the wound, and protect the smooth healing of the wound (Obagi et al., 2019). However, skin wound healing is a continuous and dynamic process, during which neutrophils, fibroblasts, epithelial cells, growth factors, cytokines and other cells and factors interact to regulate (Velnar et al., 2009; Golebiewska and Poole, 2015). At the same time, in the process of wound healing, it is also necessary to maintain a sterile, breathable, moist stable and appropriate microenvironment to ensure the smooth evolution of each stage of the whole healing process (Pereira and Bártolo, 2016; Wang et al., 2018). The wound dressing formed by a single or a combination of two matrix materials cannot meet many requirements for skin healing. In order to solve this problem, in addition to re-designing matrix materials with more comprehensive and excellent performance, it has become a feasible and effective method to use growth factors or nanomaterials to modify matrix materials to improve the overall performance of wound dressings.

**SCHEME 1:**
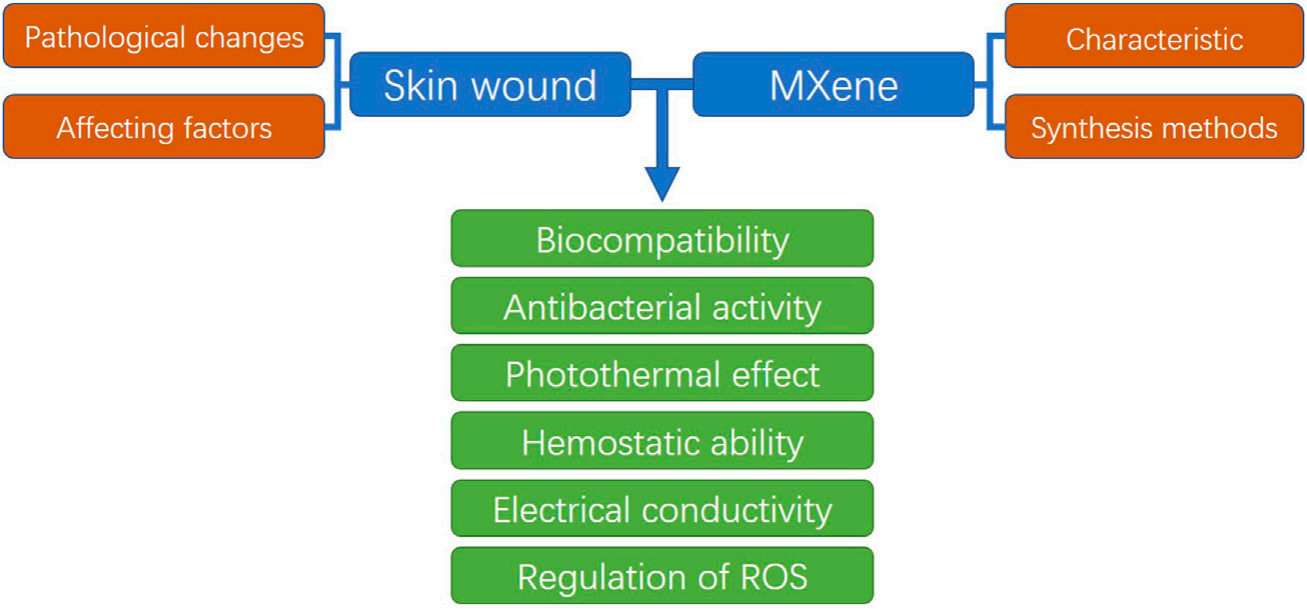
Schematic diagram of this review.

MXene is a new kind of metallic nitrogen and carbon compound, which has a two-dimensional lamellar structure similar to graphene and black phosphorus (Naguib et al., 2012). The abundant functional groups on its surface give it more abundant physical and chemical properties and biological properties. MXene has good biocompatibility, electrical conductivity and mechanical properties, but also can produce photothermal effect under NIR conditions, which makes it widely used in biological fields such as biosensing, tumor therapy, tracer imaging and so on (Naguib et al., 2012; Carey and Barsoum, 2021; Chen et al., 2021; Fadahunsi et al., 2022). In addition, researchers also found that MXene has good antibacterial activity and certain scavenging ability of active oxygen species (Jastrzębska et al., 2017; Wang et al., 2023). These biological characteristics are highly consistent with the properties required by wound dressings, making MXene become a hot material for modification of skin wound dressings in recent years (Li et al., 2022a; Liu et al., 2022a; Yang et al., 2022a). However, there has been no systematic review on the mechanism and application of MXene in skin wound repair. In this paper, we will introduce the preparation and modification methods of the emerging material MXene based on the pathophysiological changes of skin wound healing and the properties of the ideal wound dressing, and systematically review the role and application of MXene in the process of skin wound healing (Scheme 1). To provide reference for further application of MXene in skin wound dressing design by subsequent researchers.

## 2 Characteristics and properties of wounds healing

### 2.1 Normal skin structure and function

Skin is the largest organ of the human body. The skin is composed of epidermis, dermis and subcutaneous tissue from the outside to inside. According to the differentiation stage and characteristics of keratinocytes, the epidermis can be divided into four layers, from deep to shallow, which are basal layer, spinous layer, granular layer and stratum corneum. The basal layer is located at the bottom of the epidermis and consists of a cylindrical layer of epidermal stem cells, also known as basal cells. These basal cells have the ability to proliferate and differentiate, and can maintain the stability of their numbers (Prost-Squarcioni, 2006; Li et al., 2010). Spinous cells have strong protein synthesis function and can synthesize a large amount of keratin and lamellar granules. Keratinoid also distributed in the upper cells of the spinous layer, which together with the intercellular desmosomes can closely connect the spinous cells and prevent the entry of external water, thus providing protection and isolation (Wertz, 2018).

The cells in the granulosa layer are supplemented by the spinous cells in the upper part of the spinous layer. When the cells in the granulosa layer migrate to the stratum corneum, almost all the cellular structures in the cells are destroyed, and the cells turn into keratinocytes. The stratum corneum, at the top of the epidermis, is composed of protein-rich keratinocytes and the extracellular lipids that surround them. The stratum corneum is an important functional layer for the skin to resist mechanical damage, prevent water loss and environmental soluble substances from penetrating the skin (Jiao et al., 2022). In addition, there is a zona pellucida composed of 2-3 layers of flattened cells between the granular layer and the cuticle layer in the palm and plantar, called the pellucida (Elias, 2012). Based on the basal cells in the basal layer, the cells in the whole epidermis migrate outward continuously through the proliferation and differentiation of the basal cells to provide supplement for the cells in each layer and realize self-renewal and self-repair to a certain extent.

The dermis is mainly composed of connective tissue that contains nerves, blood vessels, lymphatics, muscles, and skin appendages (Woodley, 2017). The dermis can be divided into papillary layer and reticular layer from shallow to deep. The papillary layer protrudes outwards to the epidermal layer and contains rich capillaries and nerve endings, which can provide adequate nutrition for the epidermal layer (Arda et al., 2014). The mesh layer contains a large number of collagen fibers and elastic fibers. The interwoven fiber tissues provide toughness and elasticity for the skin, ensure that the skin has a certain mechanical strength and toughness, and play a protective role for the tissues and organs in the body (Usansky et al., 2021).

### 2.2 Pathophysiological changes of skin wound healing

**FIGURE 1:**
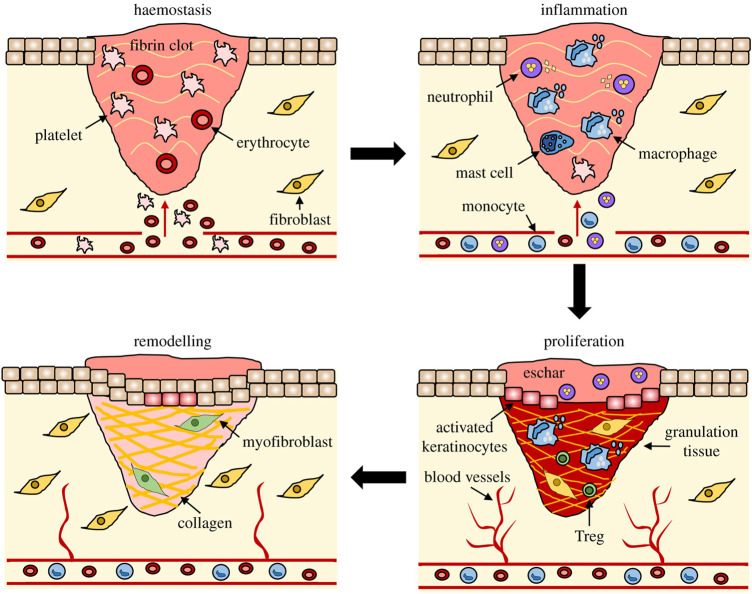
The stages of wound repair and their major cellular components (Wilkinson and Hardman, 2020).

After skin injury occurs, the body completes skin wound repair through a series of continuous pathophysiological changes. The whole process can be roughly divided into four stages as shown in Figure 1: hemostatic stage, inflammatory stage, proliferative stage and remodeling stage (Wilkinson and Hardman, 2020). After the occurrence of skin injury, the capillaries and arteriolar arteries in the injured area are broken, and the exposed vascular endothelial cells and the foreign substances causing the injury jointly activate the internal and external coagulation cascade, prompting platelet activation and accumulation to the injured site (Velnar et al., 2009). Through the release of endogenous ADP and thromboxane A2 in the platelets, the platelets undergo irreversible coagulation and form platelet thrombosis (Golebiewska and Poole, 2015). Platelet thrombus, together with fibrin, fibronectin, further constitute insoluble clots that act as wound packing and hemostasis (Broughton et al., 2006). In addition, clots composed of platelets and proteins can also provide attachment scaffolds for immune cells, release a variety of cytokines and inflammatory factors, promote the migration and aggregation of inflammatory cells and activate inflammatory response (Cooke, 2019).

Under the induction of inflammatory factors, neutrophils first gather to the injured area, phagocytosis and release reactive oxygen species, antimicrobial peptides, proteolytic enzymes to engulf and remove necrotic tissues and pathogens (Li et al., 2007). Neutrophils also continue to release pro-inflammatory factors, further stimulating the aggregation of neutrophils and macrophages to the injured area (Paquet and Piérard, 1996; Rodero and Khosrotehrani, 2010). With the removal of necrotic tissue and pathogens from the injured area, the number of neutrophils gradually decreases. Most neutrophils are squeezed out from the wound area, and the remaining neutrophils are gradually removed by recruited macrophages through endocytosis (SingerHealing, 2022). As the inflammatory response progresses, macrophages shift from a pro-inflammatory phenotype to an anti-inflammatory phenotype at the end of inflammation by releasing a variety of growth factors that promote angiogenesis, fibroplasia, and skin re-epithelialization (Hunt et al., 2000).

When the wound repair entered the proliferative stage, keratinocytes, fibroblasts and endothelial cells began to proliferate under the action of EGF, FGF, VEGF and other growth factors (Werner and Grose, 2003; Lichtman et al., 2016). The keratinocytes at the wound edge become more polar and migratory and begin to migrate to the injured area where they proliferate and differentiate to form a new upper layer, which known as re-epithelialization (Rousselle et al., 2019). At the same time, fibroblasts synthesize a large amount of type III collagen, proteoglycan and fibronectin to form extracellular matrix, which provides skeleton structure for cell migration and proliferation to the injured area (Bártolo et al., 2022). Under the action of growth factors such as VEGF, endothelial cells migrate to the injured area and proliferate to form a new capillary network (Park et al., 2017). Together with the newly generated extracellular matrix and keratinocytes in the wound, constitute granulation tissue.

Wound healing begins to enter the remodeling stage at 2–3 weeks after injury, which is mainly the remodeling of new tissue and the formation of scar tissue. The new granulation tissue is mainly composed of type III collagen with low elastic tension, while the normal skin tissue is mainly composed of type I collagen with higher tensile strength (Rippa et al., 2019; Wikslund et al., 2022). Therefore, under the action of fibroblasts and collagenase, collagen in granulation tissue is constantly degraded and regenerated to form higher strength type I collagen, thus providing scar tissue with mechanical strength close to that of normal skin tissue (Churko and Laird, 2013). At the same time, the excess capillaries and residual inflammatory cells formed in the repair process will be gradually eliminated by apoptosis, and eventually scar tissue will be formed (Kimura and Tsuji, 2021).

### 2.3 Related factors affecting skin wound healing

Under physiological conditions, the wound repair process can be completed in about 2 weeks. The factors affecting wound healing can be divided into endogenous factors and exogenous factors according to different sources (Table 1). Common endogenous factors include age, nutritional status, physical health status, hormone level and genetic factors (Winter 2006). With the increase of age, the proliferation activity of keratinocytes and basal cells in the epidermis of the skin decreases, so it is more prone to damage under the influence of external injuries (Boismal et al., 2020). At the same time, the decrease in the activity of macrophages and the decrease in the release of growth factors and cytokines caused by aging prolongs the time of proliferation, which leads to the prolongation of the wound healing process and the increase of the time required (Bonté et al., 2019).

**TABLE 1:** Factors affecting skin wound healing.

Endogenous factors	Exogenous factors
Age	Drinking
Hormone	Infection
Nutrition	Smoking
Chronic disease	
Heredity	

In addition to the slowing down of tissue renewal caused by aging, the health status of the body also affects wound healing. Some chronic diseases such as diabetes can affect the abnormal formation of micro vessels during wound healing, resulting in delayed skin re-epithelialization and abnormal extracellular matrix remodeling. Vitamins, essential fatty acids and other nutrients also play a key role in wound healing. Lack of these nutrients can lead to prolonged wound healing and an increased risk of infection (Pullar et al., 2017). Proper supplements of vitamins and fatty acids such as vitamin A, vitamin C and n-3 fatty acids can speed up wound healing (Huang et al., 2018a; VanBuren and Everts, 2022). Hormones also have a certain impact on the healing of skin wounds. Glucocorticoid can inhibit the inflammatory response, slow down the aggregation of neutrophils and macrophages to the wound during the inflammatory period, and prolong the wound healing time (Hengge et al., 2006). Estrogen can promote the re-epithelialization of keratinocytes and angiogenesis of endothelial cells, and accelerate wound healing (Wilkinson and Hardman, 2017). Insulin controls blood sugar levels to achieve normal wound healing, avoiding microvascular abnormalities caused by hyperglycemia and energy supply disorders caused by hypoglycemia (Hrynyk and Neufeld, 2014; Yu et al., 2019). Finally, wound healing is also affected by genetic factors. For example, people with cicatricial constitution may produce excessive scar of wound fiber due to excessive deposition of collagen, thus forming scar healing (Amadeu et al., 2004).

In addition to endogenous factors, exogenous factors also have significant influence on the healing of skin wounds. When the bacteria in the environment come into contact with the wound, the bacteria will gather and grow on the wound, release toxins and cause the necrosis of tissues and cells (Zulkowski, 2013). In the process of removing bacteria, inflammatory factors will be released excessively, resulting in the imbalance between growth factors and inflammatory factors, the inhibition of cell proliferation, and the delay of wound healing or prolonged wound healing (Scalise et al., 2015; Malone and Schultz, 2022). Smoking also has an obvious adverse effect on wound healing. Nicotine and NO in cigarettes can cause small blood vessel constriction, increase platelet adhesion, cause small blood vessel occlusion (Ortiz and Grando, 2012). Alcohol inhibits the body’s immune response while reducing the level of collagen forming MMPs, which affects the normal healing of wound (Rosa et al., 2018).

## 3 Characteristics of wound dressing in skin wound healing

### 3.1 Types and main functions of wound dressings

As a kind of open wound, the external environment has obvious influence on the healing process of skin wound (Kruse et al., 2015; Kirchner et al., 2020). Early wound dressing is mainly made of gauze, cotton and other materials, applied to the wound can quickly stop bleeding, absorb exudation, help the wound drainage, reduce the chance of wound infection (Pereira and Bártolo, 2016; Farahani and Shafiee, 2021). However, these traditional wound dressings can not effectively maintain the moist wound environment (Aljghami et al., 2019). At the same time, there are still large pores in these traditional dressings, which cannot avoid the contact between bacteria in the air and the wound (Simões et al., 2018; Obagi et al., 2019). With the development of time, modern dressings represented by hydrogels, fiber dressings, foam dressings and film dressings are gradually applied in clinical practice (Walker et al., 2017; Liang et al., 2021a; Tan et al., 2022). These dressings can create a moist surface environment for the wound and prevent bacteria from passing through the dressing and entering the wound while ensuring gas exchange (Francesko et al., 2018). Fiber dressings such as alginate fiber dressings also have excellent absorbency and are able to fully absorb the wound exudation, keeping the wound relatively dry (Zhang and Zhao, 2020). Since wound healing is affected by many internal and external factors, it has become a new direction for the construction of wound dressings to modify or add a variety of bioactive substances to the existing matrix materials and make them have anti-inflammatory, antibacterial, promoting re-epithelialization and other biological functions (Zhao et al., 2017; Zhang et al., 2022a; Yao et al., 2023).

### 3.2 Properties of an ideal wound dressing

Although the properties of dressings is improving, there is still a certain gap compared with the healing effect of autologous skin transplantation (Herskovitz et al., 2016). An ideal wound dressing should be able to meet the needs of all aspects of the wound healing process and provide the most suitable internal and external environment for cell and tissue regeneration. Firstly, the dressing must have good biocompatibility in the selection of raw materials, and will not cause immune rejection or biological toxicity (Zhang et al., 2022a); At the same time, it should have relatively low economic cost, which is convenient for large-scale production and clinical application (Pagnamenta, 2017). The wound dressing constructed should be able to simulate the tissue structure of the skin, have appropriate mechanical strength, and be able to fit closely with the skin without adhesion to the wound, so as to avoid the occurrence of secondary damage (Alizadehgiashi et al., 2021). In addition, the wound dressing should also have good moisture, air permeability and water absorption, can fully absorb the wound exudate, to ensure the gas exchange between the wound and the outside world (Liang et al., 2021b; Yang et al., 2022a). Finally, the wound dressing should have a certain antibacterial and bactericidal ability, to minimize the occurrence of wound infection; And on this basis, it has the ability to promote cell proliferation and growth and skin regeneration (Jiang and Loo, 2021; Yuan et al., 2022). Obviously, a single kind of material cannot meet all the above needs. Therefore, it will be a future research trend to construct bioactive materials and tissue engineering dressings by adding bioactive substances or stem cells to matrix materials through multi-material combination.

## 4 Properties and preparation of MXene

### 4.1 Characteristics of MXene

**FIGURE 2:**
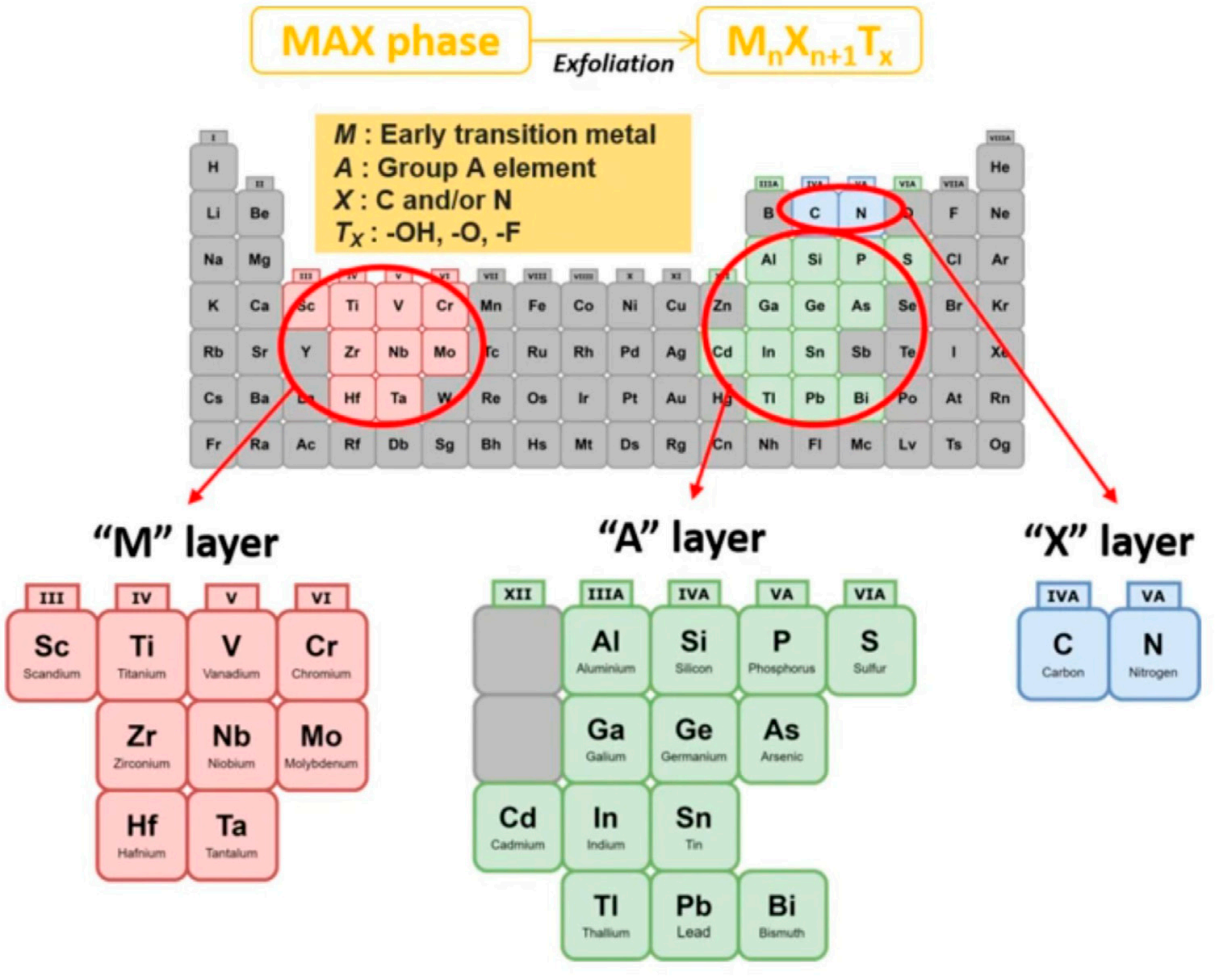
General element composition of MAX phase and MXene: M: early transition metal, A: Group A element, X: C and/or N, Tx: surface functional group (Zamhuri et al., 2021).

**FIGURE 3:**
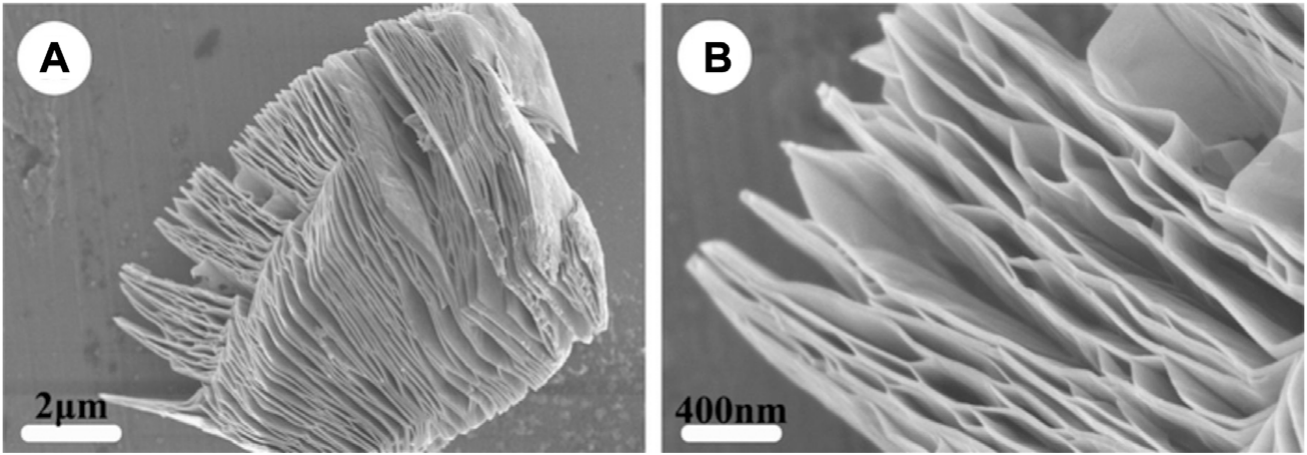
Structure of MXene. SEM images of **(A)** MXene-Ti3C2 and **(B)** the high-magnification of **(A)**. (Wang et al., 2015).

MXene is a general term for a class of two-dimensional metal carbides, whose structure is generally M_n+1_X_n_, where M represents excessive metallic elements and X represents carbon, nitrogen, or a carbon-nitrogen complex (Naguib et al., 2012). MXene is usually obtained by etching the A atomic layer in the MAX phase of its precursor. As shown in Figure 2 and Figure 3, MAX is communicated as M_n+1_AX_n_, where A represents an element of the third or fourth main group, usually Al and Si (Zamhuri et al., 2021). The M-A bond in MAX has the properties of a metallic bond with a weak force relative to the M-X bond, so the A phase in MAX can be etched out by a mixture of HF or HCl with fluorine salts, leaving the M atomic layer and X atomic layer to form two-dimensional M_n+1_X_n_. Since MXene prepared by liquid phase method has functional groups such as -OH, -O and -F, it is often written as M_n+1_X_n_T_x_, where T stands for the surface functional groups in the compound and x indicates the number of such groups (Naguib et al., 2014).

Since Naguib et al. first discovered and synthesized MXene in 2012, dozens of different MXene have been prepared and applied in many fields, such as catalysis, sensor, energy storage, microwave absorption, biomedicine and so on (Pang et al., 2019; Soleymaniha et al., 2019; Huang et al., 2021). The transition metals, surface functional groups and unique two-dimensional lamellar structure of MXene give it a rich variety of properties. MXene composed of Ti, Ta and Nb in the transition elements has good stability and biocompatibility in animals, and does not cause obvious immune response and pathological changes in the body (Sundaram et al., 2020). Surface functional groups and electronegative layered structures provide MXene with good hydrophilicity (Lu et al., 2021). Compared with two-dimensional materials such as graphene, MXene has better water dispersion and hydrophilicity, and can be uniformly dispersed in water-based solvent to construct a hydrophilic composite material with good cytocompatibility and adhesion (Chen et al., 2017; Lin et al., 2021). The abundant surface functional groups also provide a large number of binding sites for MXene, which can be combined with other matrix materials, proteins, drugs and other biological macromolecules to achieve material modification and drug delivery, greatly expanding the application of MXene in the field of biomedicine (Huang et al., 2020a; Koyappayil et al., 2022). MXene also has an electrical conductivity close to that of graphene, and can vary between semiconductors and conductors according to the modification of its surface groups, meeting different electrical conductivity requirements of materials (Yin et al., 2021a; Riazi et al., 2021). The mechanical destruction of bacterial cell membranes by MXene’s two-dimensional lamellar structure and the redox action of lipopolysaccharides by strong anions on cell membranes give MXene excellent antibacterial properties (Begum et al., 2020; Hao et al., 2022). In addition, MXene has strong light absorption in the NIR region, which makes MXene also promising in the field of photothermal therapy and imaging (Yin et al., 2021b; Jiang et al., 2022).

### 4.2 Preparation methods of MXene

#### 4.2.1 Hydrofluoric acid etching method

Etching the MAX phase using acid is the most commonly used method for preparing block MXene (Zhang et al., 2022b). Etching the Max phase using acid is the most commonly used method for preparing block MXene, and among all candidates, HF was the earliest one being applied. Based on the difference of bonding force between M-A bond and M-X bond in MAX, HF targets the M-A bond in MAX, where high concentration of HF provides fluorine ions that binds selectively and tightly to A element (He et al., 2021a; Chen et al., 2021). By adjusting HF concentration and reaction time to control the degree of reaction, MXene two-dimensional laminates prepared by different MAX phases and MXene blocks of different thickness can be obtained. For example, when 40% HF is used for etching, Ti_3_AlC_2_ powder needs to be etched for 24 h to obtain multilayer Ti_3_C_2_ nanosheets (Awasthi et al., 2020), while Nb_2_C can be prepared only by treating Nb_2_AlC powder for 3 h (He et al., 2021b). However, it should be noted that MXene prepared by etching MAX phase with HF is mostly accordion-like multilayer structure. If single-layer two-dimensional MXene sheets need to be obtained, intercalator such as DMSO should be introduced into the reaction system or ultrasonic wave should be used for delamination stripping (Naguib et al., 2015; Rajavel et al., 2018). Due to the strong corrosion of HF, it is dangerous for operators (Ozcan et al., 2012). Meanwhile, the use of high concentration HF to treat MAX phase for a long time increases the defects in the prepared MXene layer and reduces the transverse size (Ghidiu et al., 2014). Therefore, the use of HCl to replace part of HF in the reaction system has also become a feasible preparation method (Wei et al., 2021; Yu et al., 2021).

#### 4.2.2 *In situ* hydrofluoric acid etching

High safety risks exist in the preparation of MXene using HF, and additional intercalation agents are required in the preparation of monolayer two-dimensional MXene, which has led to the search for new safer and more efficient methods of MXene preparation. The substitution of M-A layer by the *in-situ* HF formation on the surface of the material by the combination of HCl and fluoride salt has become the mainstream preparation method (Baraneedharan et al., 2022; Kumar et al., 2022). In this method, LiF and HCl are usually used as etching agents. Cations in fluoride salts can also be used as intercalating agents to enable MXene to be stratified and stripped, so as to obtain single two-dimensional MXene, eliminating the need to introduce additional intercalating agents (Ghazaly et al., 2021; Sinha et al., 2021). Besides LiF, fluoride salts such as NaF, KF and NH4F exerts similar effects in the etching process (Liu et al., 2017a; Sun et al., 2023). In addition, given that neither HF nor fluoride brine solutions can substitute A layer in the preparation of nitride-based MAX, MAX is mixed with molten fluoride salt mixture in an argon atmosphere (Urbankowski et al., 2016).

#### 4.2.3 Fluorine-free preparation method

Whether HF is used for direct etching or HF *in situ* etching, HF formation is inevitably involved in the reaction process. HF is very harmful to human body, and a small amount of HF direct contact can cause the necrosis of cell tissues and even lead to death (Kaminsky et al., 1990; Miranda et al., 2021). In addition, the introduction of fluoride ions in the reaction system will reduce the number of -OH, -O and other functional groups on the surface of MXene, which is not conducive to the further modification of MXene in the application of biomedicine (Huang et al., 2018b). Therefore, the construction of a fluorine-free MXene preparation method will be more conducive to the application of MXene in the biomedical field. Yang et al. designed a method for preparing Ti_3_C_2_ nanosheets using electrochemical etching of NH_4_Cl and TMAOH (Yang et al., 2018). Two Ti_3_AlC_2_ nanosheets were used as anode and cathode respectively, during the etching process, chloride ion in the solution binds tightly to Al, consequently pure Ti_3_C_2_ is collected. Li et al. designed a NaOH-assisted hydrothermal process to prepare Ti_3_C_2_ and obtained a 92% purity Ti_3_C_2_ powder (Li et al., 2018). Since no fluoride ion is involved in the preparation process, the Ti_3_C_2_ collected in this way possess more active functional groups and have more potentiality for biological modification.

In addition to MXene obtained by treating MAX phase, MXene nanosheets can also be prepared by chemical vapor deposition (Li et al., 2021a; Thirumal et al., 2022). This method is mainly used to prepare some two-dimensional MXene that cannot be synthesized by etching or does not exist stable MAX precursor phase, such as TaC, TaN, etc (Liu et al., 2021). For example, Geng et al. used a CVD process catalyzed by molten copper to prepare Mo_2_C thin layers on graphene surface *in situ* (Geng et al., 2017). Wang et al. heated Cu and Ta with acetylene gas to prepare TaC thin nanosheets (Wang et al., 2017). In summary, the current methods of preparing MXene can be roughly divided into two categories according to whether fluorine ions are involved in the reaction system. The method of preparing MXene using HF or *in-situ* synthesis of HF is relatively simple and easy to prepare and synthesize in large quantities. However, the biological security problems brought by fluorine ions need to be carefully applied. Fluorine-free preparation method has higher biosecurity and is more environmentally friendly because fluorine ion is not involved in the reaction system. However, its preparation process is more complex, and its yield has some disadvantages compared with traditional methods.

## 5 Application of MXene and its modified materials in skin wound healing

### 5.1 Biocompatibility

As a new material, good cytocompatibility and tissue non-toxicity are the prerequisite for its further application in the biomedical field. Up to now, there are dozens of two-dimensional transition metal compounds in the MXene family, but only Ti, Nb, Ta several transition elements and their compounds with relatively stable chemical properties can be applied in the field of biomedicine, and Ti_3_C_2_ is the main application in skin wound healing materials (Jastrzębska et al., 2017; Liu et al., 2022a). For Ti_3_C_2_, its biocompatibility is affected by many factors, such as concentration, size, synthesis method and administration route (Scheibe et al., 2019; Szuplewska et al., 2019). In general, MXene has no obvious toxic and side effects on most cell lines at low and medium concentration. However, when the concentration of MXene increases gradually, the activity of tumor cell lines will be significantly decreased. When the concentration of MXene reaches 500 mg/L, normal cell lines can still maintain 70% or even higher cell activity. On the other hand, the activity of tumor cell lines decreased significantly, and only about 20% of A549 cell line still had proliferative activity at this concentration (Jastrzębska et al., 2017). This phenomenon may be due to the fact that MXene produces reactive oxygen species that exceed the oxidative stress level of cancer cells, thus leading to apoptosis of cancer cells (Hu et al., 2010).

The morphology of MXene applied in biomaterials also has some influence on its biosafety. Zhou et al. found that the safe concentration of Ti_3_C_2_ QDs for human embryonic kidney cell 2,937 and MCF-7 cancer cells could reach 400 mg/L when they selected Ti_3_C_2_ QDS as the intervention material (Zhou et al., 2017). The oxidized Ti_3_C_2_ formed by the oxidation of the functional groups on the surface of Ti_3_C_2_ showed significant cytotoxicity (Jastrzebska et al., 2020). The size of the prepared MXene also has a certain influence on its cytocompatibility. Compared with large size Ti_3_C_2_ (500 nm) under the same conditions, small size Ti_3_C_2_ (1–100 nm) showed higher activity inhibition on cells, which may be caused by the ability of small size MXene to enter cells through endocytosis and induce autophagy dysfunction (Shi et al., 2020). However, although MXene may have some effects on cell activity *in vitro*, none of the results showed any potential organ pathological changes or toxic effects when MXene was applied *in vivo in vitro*, indicating that MXene has no toxic side effects on organisms, which provides a guarantee for its safe use *in vivo* (Liu et al., 2017b; Li et al., 2021b).

**FIGURE 4:**
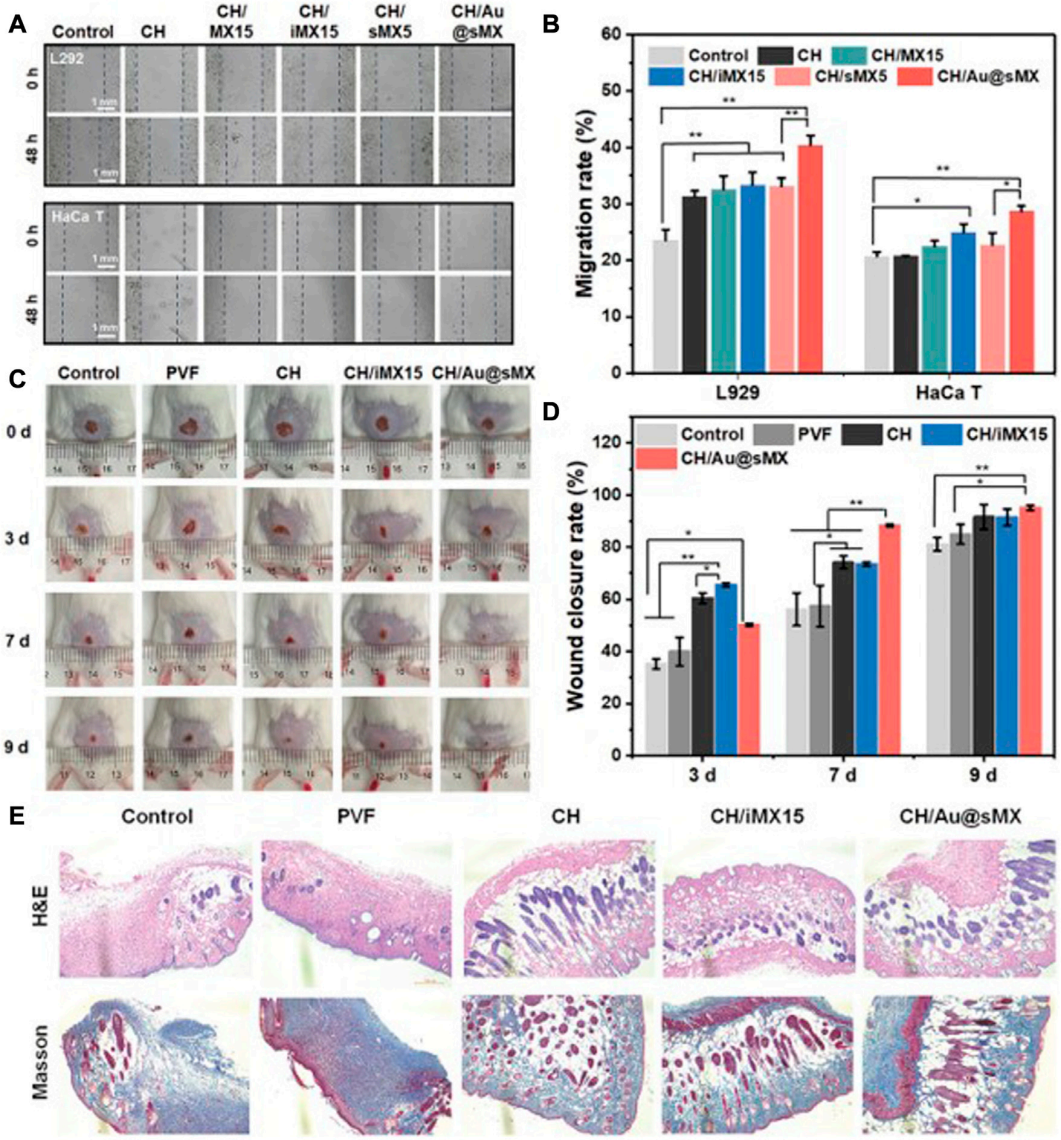
Biocompatibility of MXene-modified sponges *in vivo* and vitro **(A)** Representative images and **(B)** migration scratch assay of L292 and HaCaT at 48 and 0 h after scratching and treatment with 0.5 mg/ml of each sample. *In vivo* assessment of the sponges for wound healing **(C)** Photographic snapshots of temporal development of healing wounds for the different sponges in 0, 3, 7, and 9 days, respectively. **(D)** Wound closure rate of different sponges at different healing times **(E)** H&E staining and Masson staining images of the wound section at the 13th day for each group, respectively (Li et al., 2022b).

The process of skin wound healing is mainly realized by fibroblasts and keratinocytes. Whether MXene has toxic effects on these 2 cells determines whether it can be used in the modified design of skin wound dressings. Li et al. constructed an anisotropic MXene@PVA hydrogel in which NIH3T3 cell lines grown in the hydrogel had a cell survival rate of over 90%, in addition, NIH3T3 cells in the hydrogel showed higher cellular activity compared to the control group (Li et al., 2022a). Wang et al. constructed a SF-coated MXene membrane and showed that human skin fibroblasts HSAS1 cells were able to grow normally on the surface of the membrane and still showed 99% cellular activity after 6 days (Wang et al., 2020). These results indicated that MXene had good biosafety for fibroblasts, and did not affect the activity of fibroblasts. Li et al. inoculated HaCaT keratinocyte into MXene-containing chitin composite sponges, and Figure 4 showed that HaCaT keratinocyte migration was significantly enhanced in MXene-containing materials, with good cell survival (Li et al., 2022b). These results indicate that MXene has good cytocompatibility with the two key cells in skin injury and regeneration, and can promote fibroblast migration and wound healing to a certain extent.

### 5.2 Antibacterial activity

Wound infection is an important factor that leads to wound deterioration or delayed healing. For a wound healing dressing, it has certain antibacterial and even bactericidal properties to significantly reduce the chance of infection on the wound surface and promote the healing of the wound, especially the infected wound (Liu et al., 2022a; Li et al., 2022c; Yu et al., 2022). Traditional wound care methods for infected wounds usually use antibiotics to solve the problem of wound infection, but a wide variety of bacterial species and complex environment on the wound surface make it difficult for a single antibiotic to cover all bacterial species, and long-term use of broad-spectrum antibiotics is easy to lead to the colonization and growth of multi-drug-resistant bacteria such as MRSA, further increasing the difficulty of wound healing (Deurenberg and Stobberingh, 2008; Li et al., 2022c). MXene has a lamellar structure similar to graphene and more abundant surface groups, so the researchers hypothesized that MXene should also have some antibacterial properties. Rasool et al. investigated the antibacterial properties of MXene in 2016, and found that Ti_3_C_2_ had higher antibacterial efficiency against Gram-negative *Escherichia coli* and Gram-positive *Bacillus subtilis* compared to GO (Rasool et al., 2016). At the same time, Ti_3_C_2_ showed obvious dose-dependent bactericidal effect, and it was found that Ti_3_C_2_ could achieve 98% bactericidal killing rate at 200 μg/ml. TEM and SEM results showed that the cell membrane was destroyed under the action of Ti_3_C_2_, resulting in the release of cytoplasm from the bacteria. The authors speculate that this strong antibacterial activity may be caused by bacterial oxidative stress caused by electron transfer and the direct mechanical damage of MXene lamellar structure to the cell membrane (Mashtalir et al., 2014; Tian et al., 2014).

**FIGURE 5:**
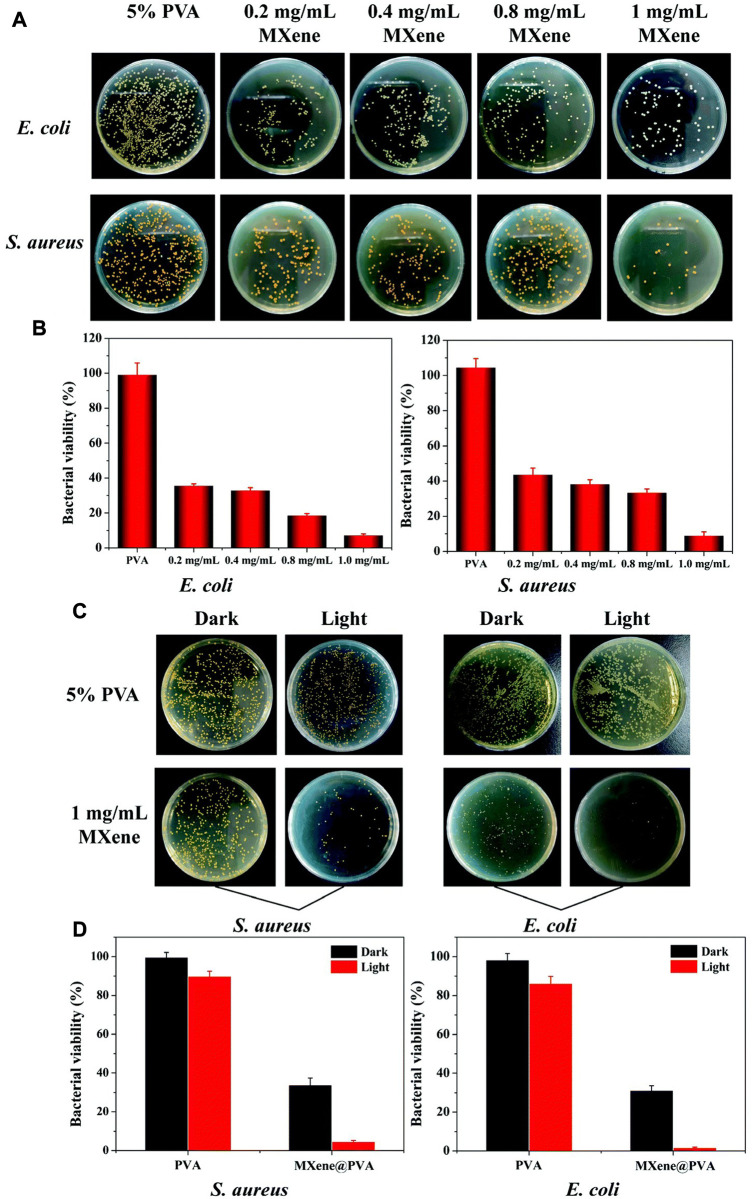
Antibacterial ability of MXene-modified MXene@PVA hydrogel **(A)** Photograph of bacterial colonies of *Escherichia coli* and *Staphylococcus aureus* treated with different concentrations of MXene. **(C)** Photograph of bacterial colonies formed by *Escherichia coli* and *Staphylococcus aureus* treated with the PVA hydrogel, the PVA hydrogel + NIR, MXene@PVA hydrogel (1 mg/ml MXene) and the MXene@PVA hydrogel (1 mg/ml MXene) + NIR. The power density was 1.5 W/cm^2^, and the operation time was 10 min **(B)**; **(D)** corresponding survival rates for *Escherichia coli* and *Staphylococcus aureus* (Li et al., 2022a).

MXene can avoid the emergence of bacterial resistance through the mechanism of bacterial death caused by mechanical damage and oxidative stress, and can also maintain a good killing effect against multi-drug resistant bacteria, which makes more researchers apply MXene in the modification and construction of wound dressings (Figure 5). Mayerberger, E. A. et al. constructed a chitosan nanofiber loaded with MXene. When *E. coli* and *Staphylococcus aureus* were inoculated on the fibers, they showed a 95% and 62% reduction in the number of colonies formed, respectively, 4 hours after culture (Mayerberger et al., 2018). Rozmysłowska-Wojciechowska et al. constructed a MXene modified chitosan-hyaluronic acid hydrogel and demonstrated up to 90% growth inhibition against *E. coli* and *S. aureus* in hydrogels supplemented with only 1% MXene (Rozmysłowska-Wojciechowska et al., 2020). In addition to the antibacterial effect of MXene itself, the abundant functional groups on the surface of MXene give it great potential for modification, which can be combined with proteins, growth factors, nanoparticles and other molecules to play more roles. Zhang et al. fixed lysozyme onto the surface of MXene nanosheets. This composite demonstrated excellent MRSA killing ability and significantly promoted the healing of infected wound (Zhang et al., 2023).

### 5.3 Photothermal effect

Photothermal effect refers to the phenomenon of increasing temperature caused by the interaction between photon energy and lattice vibration after the material is irradiated by light, among which the photothermal effect caused by near-infrared radiation is the most obvious (Huang et al., 2020b; Meng et al., 2022). It is found that MXene has high photothermal conversion efficiency, and can achieve obvious temperature increase under the condition of low power NIR irradiation (Xu et al., 2020; Safaei and Shishehbore, 2021). Jin et al. found that for the constructed nanofiber hydrogel loaded with MXene, 0.5 W NIR irradiated for 5min could increase the material temperature from 23°C to 41°C, and the material temperature further rose to 61°C after 5 min irradiated with 1 W NIR (Jin et al., 2021). Such excellent photothermal effect provides a new design and application idea for the application of MXene in the design of skin wound dressing. On the one hand, MXene can make the material temperature rise to a higher temperature through the photothermal effect, at 40°C or higher temperature can significantly inhibit the growth activity of bacteria, and further enhance the antibacterial and bactericidal effect of the material together with the mechanical damage effect of MXene on bacteria. Wang et al. constructed a chitosan-MXene suspension and loaded it on PVDF membrane to form a multifunctional membrane. The antibacterial experiment results showed that the antibacterial ability of the multifunctional membrane combined with NIR was close to 100%, which was significantly improved compared with the simple material group without NIR irradiation (Wang et al., 2022). The results of animal experiments showed that on the 14th day of treatment, the healing rate of the infected wound reached 95% in the MXene combined with NIR irradiation group, which was higher than that in the material group without NIR irradiation. These results indicated that the photothermal effect of MXene could further enhance the antibacterial properties of MXene and promote the healing effect of MXene on infected wounds.

**FIGURE 6:**
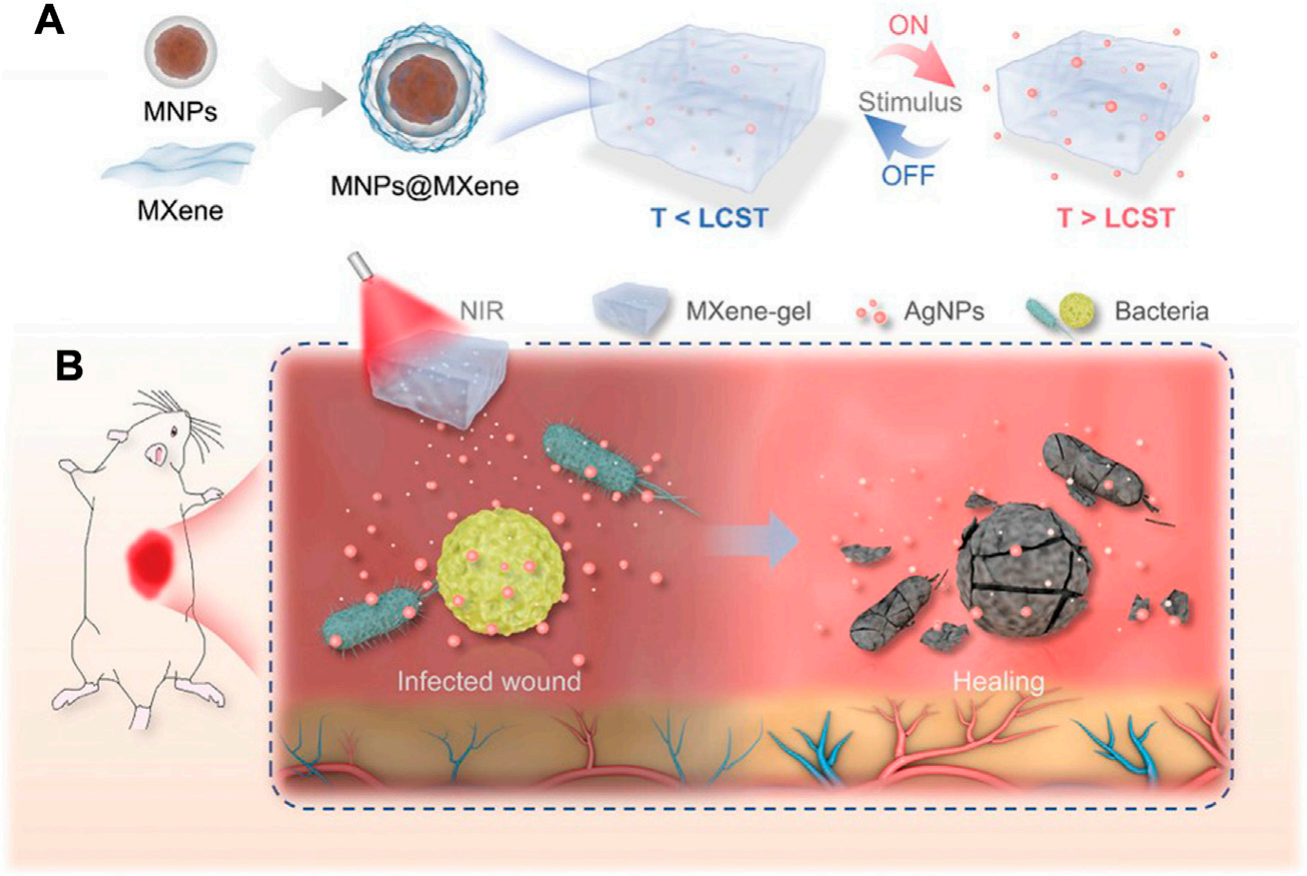
Schematic illustration of the preparation and application of NIR-responsive MXene-based hydrogel system. **(A)** The formation and drug release process of the MXene-based hydrogel system. **(B)** Deep chronic infected wound treated with NIR responsive AgNPs-loaded MXene-based hydrogel system. (Yang et al., 2022b).

On the other hand, the photothermal effect of MXene can be used to regulate the properties of the materials, so as to realize the on-demand release and precise regulation of growth factors or other active substances in the materials. Yang et al. constructed a multi-stimulus response MXene@AgNPs hydrogel (Figure 6), which achieved precise controlled release of AgNPs through photo response and magnetic response, and avoided the cytotoxicity caused by high concentration of AgNPs in a short time while guaranteeing the bactericidal effect (Yang et al., 2022b). The results of animal experiments showed that the elevation of local temperature after NIR irradiation further enhanced the action depth of AgNPs and improved the therapeutic effect of the material on deep chronic infected wounds. Xu et al. constructed a multi-mode antibacterial platform based on MXene, which utilized the photothermal effect of MXene to achieve continuous and stable release of amoxicillin, and achieved better long-term antibacterial effect under the condition of low drug loading (Xu et al., 2021). Jin et al. constructed a temperature-responsive MXene nanoribbon loaded with vitamin E. The surface temperature of the material was raised by NIR irradiation to realize the dissolution and release of vitamin E, which effectively improved the wound healing function of the material (Jin et al., 2021).

### 5.4 Hemostatic ability

**FIGURE 7:**
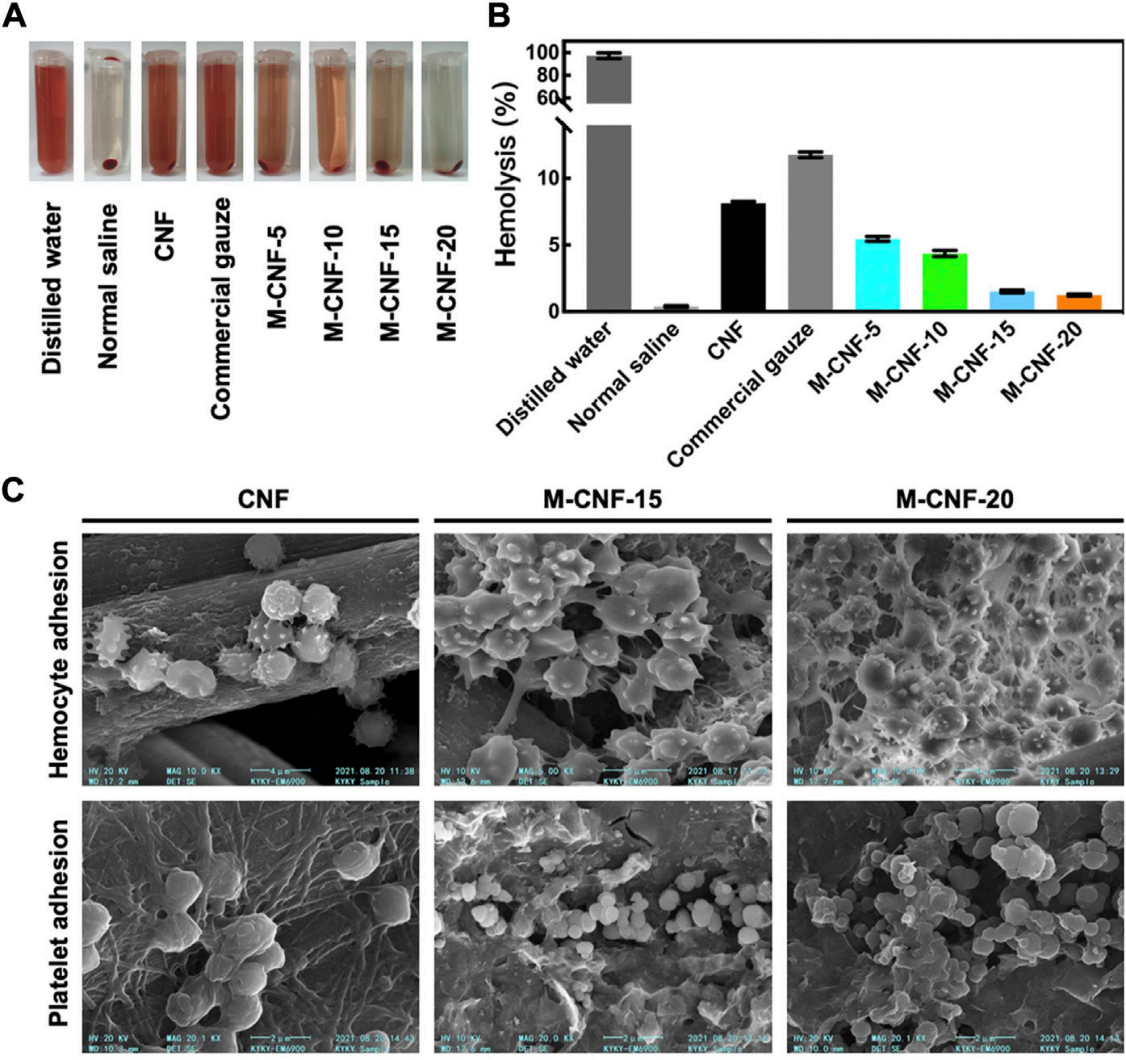
Hemostatic ability of MXene modified material M-CNF **(A, B)** Hemolysis image and hemolysis ratios of the M-CNF-x extracts. **(C)** SEM images of blood cells and platelets adhesion on the CNF, M-CNF-15 and M-CNF-20 surface (Li et al., 2022d).

Blood vessels rupture after skin injury occurs, and rapid hemostasis in the early stage of injury to form clots is conducive to wound healing and reduce the risk of infection (Guo and Dipietro, 2010; Rodriguez-Merchan, 2012). MXene has a rich surface charge that promotes blood cell aggregation, activates platelet activation and clot formation (Liu et al., 2018; Wu et al., 2021). Li et al. constructed a MXene@PDA decorated chitosan nanofiber wound dressing, which was shown to have safe blood compatibility and induce higher blood cell and platelet adhesion *in vivo* and *in vitro* (Figure 7). The authors speculate that this is mainly due to the large number of hydroxyl groups in MXene@PDA, which can adhere to blood cells and platelets and induce blood cell aggregation, platelet activation, and clot formation through interaction with plasma fibrin (Li et al., 2022d). Zhou et al. constructed a conductive antibacterial hemostatic multifunctional scaffold based on MXene nanosheets to promote wound healing in multidrug-resistant bacterial infections. The results showed that the MXene modified HPEM scaffold could significantly reduce the amount of wound bleeding, shorten the coagulation time, and demonstrate excellent hemostasis and coagulation ability. At the same time, the HPEM scaffold significantly accelerated the wound healing of MRSA-infected by effective anti-inflammatory effects, promoting cell proliferation and angiogenesis, stimulating granulation tissue formation, collagen deposition, vascular endothelial differentiation and angiogenesis (Zhou et al., 2021). Yang et al. designed a PCL-based MX@AgP bio-HJs antibacterial fiber membrane, which can realize sterilization, hemostasis, promote re-epithelialization and collagen deposition with the aid of NIR, providing a good regeneration microenvironment for wounds and promoting the healing of infected wounds (Yang et al., 2022c).

### 5.5 Electrical conductivity

Healthy skin can form a kind of "skin battery” function (Foulds and Barker, 1983; McCaig et al., 2005). When the skin is damaged to form a wound, the normal epithelial potential is immediately short-circuited, and the current flows out from the center of the wound to form a relatively stable current circuit at the edge of the wound, which is called the damaging endogenous electric field (Luo et al., 2021). A large number of studies have proved that the injurious endogenous electric field plays a vital role in the healing of skin injury (Isseroff and Dahle, 2012; Ashrafi et al., 2017; Verdes et al., 2022). As an exogenous electric field, bioelectrical stimulation can stimulate macrophages, lymphocytes and neutrophils to migrate to the wound in the early stage of skin wound healing (Demir et al., 2004), reduce the number of immune cells and cytokines in the late stage of healing (Weiss et al., 1989), increase the local tissue blood flow (Taşkan et al., 1997), reduce edema reaction (Gürgen et al., 2014), promote the migration and proliferation of fibroblasts and epithelial cells (Asadi et al., 2013), and accelerate the wound healing process (Franklin et al., 2016). Tang et al. constructed a chitosan-fibroin protein scaffold containing GO, which has good electrical conductivity. The experimental results showed that this scaffold combined with electrical stimulation could significantly improve the migration and proliferation of fibroblasts, and promote the healing of skin wounds (Tang et al., 2019).

**FIGURE 8:**
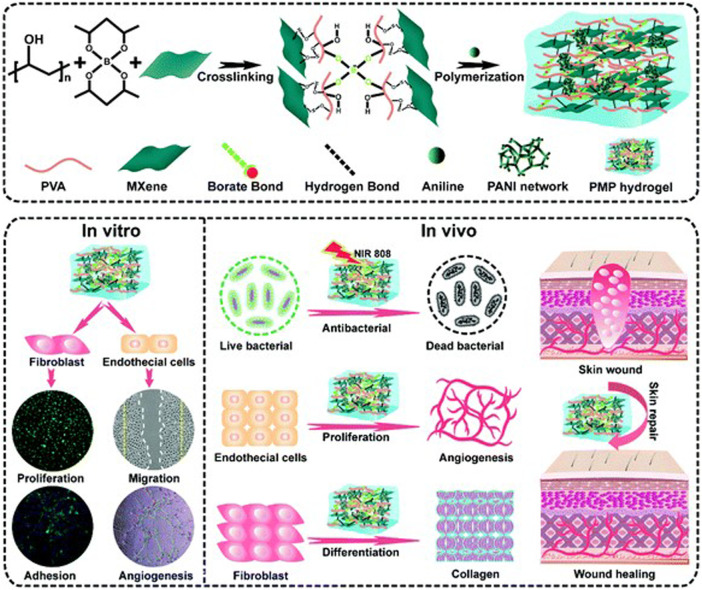
Schematic illustration of the fabrication of MXene-modified PMP hydrogels and their application in skin wound healing (Liu et al., 2022b).

As a two-dimensional nanosheet with similar structure to GO, MXene is endowed with good electrical conductivity by a large number of delocalized electrons. Mao et al. constructed a bacterial cellulose composite hydrogel containing MXene, which had good electrical and mechanical properties. The results of *in vitro* and *in vivo* experiments showed that the combination of hydrogel and electrical stimulation could significantly enhance the proliferation activity of NIH3T3 cells and accelerate the wound healing process compared with the group without electrical stimulation (Mao et al., 2020). Zhu et al. constructed an electroactive oxidized alginate/gelatin/MXene composite hydrogel. Compared with alginate/gelatin hydrogel alone, this composite material showed better mechanical properties and electroactivity. Meanwhile, it had good cytocompatibility to NIH3T3 cells and could promote the attachment and migration of fibroblasts (Zhu et al., 2022). Zheng et al. developed an injectable multifunctional hydrogel scaffold based on MXene@CeO2 nanocomposite material, which has good electrical conductivity, antioxidant capacity, antibacterial properties and mechanical properties. The multifunctional hydrogel can significantly promote the proliferation and migration of fibroblasts under the intervention of electrical stimulation. Animal experimental results showed that the composite significantly accelerated MDR wound healing by promoting granulation tissue formation, collagen deposition and re-epithelialization (Zheng et al., 2021). Liu et al. constructed a high-strength, conductive and antibacterial PVA hydrogel containing MXene and polyaniline (Figure 8). MXene in the hydrogel irradiated by NIR provided good antibacterial properties, while MXene and PANI endowed the material with excellent electrical conductivity. Applying electrical stimulation to the fibroblasts in the hydrogel can significantly promote their proliferation and migration. Animal experiments have shown that this multifunctional hydrogel can significantly accelerate skin wound healing by promoting angiogenesis and collagen deposition (Liu et al., 2022b).

### 5.6 Regulation of reactive oxygen species and inflammatory responses

In the process of skin wound healing, the local inflammatory response of the injury is in a dynamic balance. After injury, the local inflammatory response formed in the early stage of injury and the release of cytokines are conducive to the migration of macrophages, neutrophils and lymphocytes to the injured area, and accelerate the removal of wound cell debris and foreign substances (Golebiewska and Poole, 2015). When entering the proliferative and remodeling stages, a large number of inflammatory cells gathered in the injured area are gradually replaced by transplanted fibroblasts and epithelial cells, while the level of cytokines gradually decreases and various growth factors gradually increase, resulting in the formation of granulation tissue in the wound area, and the final healing is realized through remodeling (Dunnill et al., 2017; Deng et al., 2021). However, many factors such as diabetes, bacterial infection and other factors can interfere this immune response balance, which is characterized by excessive production of reactive oxygen species, pro-inflammatory cytokines and protease (Huang et al., 2020c; Tu et al., 2022). Excess ROS can cause oxidative damage to cells and tissues, inhibiting angiogenesis, granulation tissue formation, and wound healing (Wang et al., 2023). The use of wound dressings with reactive oxygen scavenging ability can reduce the inflammatory response of the wound and accelerate wound healing (Li et al., 2021c; Li et al., 2022e). MXene contains a large number of delocalized electrons, which can realize rapid conduction and delivery of electrons (Iravani et al., 2022). Meanwhile, the rich functional groups on the surface of MXene can also react with reactive oxygen species, so it has an excellent application prospect in reactive oxygen species scavenging (Iravani and Varma, 2022). Adding MXene to skin wound dressing can improve the overall scavenging ability of the material on reactive oxygen species.

**FIGURE 9:**
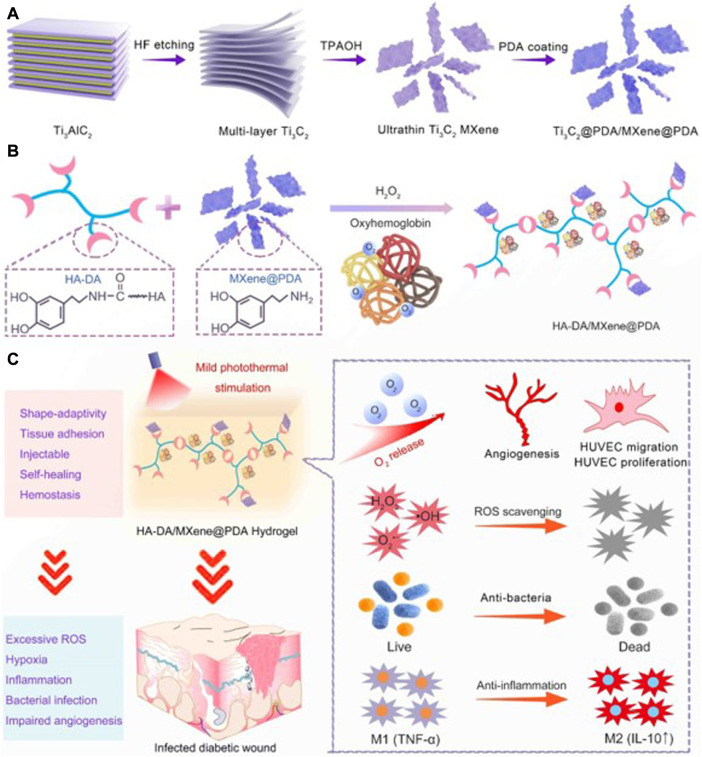
Schematic illustration of MXene@PDA Nanosheets. **(A)** Synthesis Diagram of MXene@PDA Nanosheets. **(B)** Schematic Illustrations of Injectable HA-DA/MXene@PDA Hydrogel Preparation. **(C)** Infected Diabetic Wound Healing Mechanism of HA-DA/MXene@PDA Hydrogel through Supplying O2, Scavenging ROS, Eradicating Bacteria, and Inhibiting Inflammation. (Li et al., 2022f).

Chen et al. constructed a temperature-sensitive Nb_2_C hydrogel with antioxidant and antibacterial activities. The Nb_2_C in this hydrogel can effectively remove reactive oxygen species and reduce oxidative damage of cells. Meanwhile, the hydrogel under NIR irradiation also has good antibacterial properties and can promote diabetic wound healing (Chen et al., 2022). Wei et al. constructed a new PAAM-based spongy macroporous hydrogel, in which MXene added to the hydrogel provided excellent antibacterial properties and active oxygen scavenging ability. The reactive oxygen scavenging rate of hydrogels containing MXene was up to 96% at 2 h. The authors speculated that this was mainly due to the removal of ROS by antioxidant phenol quinone groups in the MADA chain through redox reaction, and the removal effect was further enhanced by the electron transport capacity of MXene (Wei et al., 2022). Li et al. developed an injectable hydrogel based on HA-DA (Figure 9). MXene in the hydrogel showed excellent scavenging ability of reactive oxygen species and active nitrogen species, which could effectively reduce cellular inflammatory response and release of inflammatory factors, and promote the healing of diabetic infected wound (Li et al., 2022f).

## 6 Conclusion and perspectives

Skin wound healing is a continuous and complex dynamic process, which requires the joint action of a variety of cells, factors and appropriate microenvironment to achieve rapid and satisfactory healing. In order to give wound dressing a more comprehensive performance, as far as possible to meet the various needs of wound healing, the use of a variety of matrix materials combined with or add other materials to the matrix material for modification has become the main direction of wound dressing design. The rich physical and biological properties of MXene provide a good prospect for its application in the design and manufacture of wound dressing.

MXene represented by Ti_3_C_2_ has good biocompatibility. Adding MXene to the material can improve the overall mechanical properties and hemostatic ability of the material. The unique two-dimensional lamellar structure and abundant functional groups on the surface of MXene also give it excellent antibacterial properties, electrical conductivity and reactive oxygen scavenging ability, which can be further enhanced under the action of NIR. The rich functional groups on the surface of MXene also provide the potential for further modification, which further broadens the prospect of application in the design of materials for skin wound healing. The addition of MXene to wound dressings can significantly improve the overall performance of the material, giving the dressings antibacterial ability, electrical conductivity and reactive oxygen scavenging ability, and other properties conducive to skin wound healing

However, although MXene has many advantages in the application of skin wound healing and repair, there are still some shortcomings that need to be solved or improved. The existing preparation methods of MXene need to introduce hydrofluoric acid or its salt compounds, which has certain biosafety risks. When preparing MXene on a large scale, how to effectively ensure the homogeneity of the product for large-scale production and use is also a problem to be solved in the future. Although there are some potential problems in the large-scale preparation of MXene and the maintenance of the homogeneity of lamellar structure, we believe that with the continuous development of research, these problems will be able to find effective solutions, making MXene an ideal modified material for skin wound repair.
